# Experimental Gas-Phase Removal of OH Radicals in the Presence of NH_2_C(O)H over the 11.7–353 K Range: Implications in the Chemistry of the Interstellar Medium and the Earth’s Atmosphere

**DOI:** 10.1021/acsearthspacechem.4c00082

**Published:** 2024-09-16

**Authors:** Daniel González, Sara Espinosa, María Antiñolo, Marcelino Agúndez, José Cernicharo, Sydney Willis, Robin T. Garrod, Elena Jiménez

**Affiliations:** † Departamento de Química Física, Facultad de Ciencias y Tecnologías Químicas, Universidad de Castilla-La Mancha, Avda. Camilo José Cela 1B, 13071 Ciudad Real, Spain; ‡ Instituto de Investigación en Combustión y Contaminación Atmosférica, Universidad de Castilla-La Mancha, Camino de Moledores s/n, 13071 Ciudad Real, Spain; § Molecular Astrophysics Group, Instituto de Física Fundamental. Consejo Superior de Investigaciones Científicas (IFF-CSIC), C/Serrano 123, 28006 Madrid, Spain; ∥ Departments of Chemistry and Astronomy, University of Virginia, Charlottesville, Virginia 22904, United States

**Keywords:** laser techniques, supersonic expansion, ISM, gas-phase reactivity, troposphere chemistry, atmospheric lifetime, astrochemical models

## Abstract

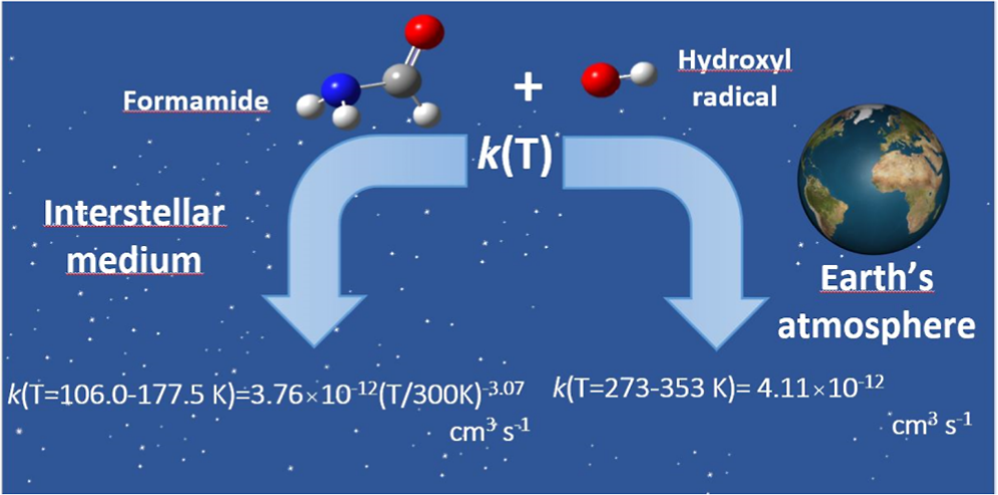

Formamide (NH_2_C(O)H) has been observed both in the interstellar medium (ISM), being identified as a potential precursor of prebiotic molecules in space, and in the Earth’s atmosphere. In these environments where temperature is very distinct, hydroxyl (OH) radicals may play an important role in the degradation of NH_2_C(O)H. Thus, in this work, we report for the first time the experimental study of the temperature dependence of the gas-phase removal of OH in the presence of NH_2_C(O)H over the 11.7–353 K range. In the lowest temperature range (11.7–177.5 K), of interest for the ISM chemistry, the kinetic study was performed using a pulsed CRESU (French acronym for Reaction Kinetics in a Uniform Supersonic Flow) apparatus, while a thermostatized slow-flow reactor was employed in the kinetic study of the OH + NH_2_C(O)H reaction over the 273–353 K range, of interest in the Earth’s troposphere below room temperature. The pulsed laser photolysis at 248 nm of a suitable OH-precursor (hydrogen peroxide, *tert*-butyl hydroperoxide, or acetylacetone) was used to generate OH radicals in the reactor. The temporal evolution of OH was monitored by laser-induced fluorescence at 310 nm. An almost independent *k*(*T*) between 273 and 353 K (temperatures of the Earth’s troposphere extended to *T* > 298 K) is reported, being the OH + NH_2_C(O)H reaction the major degradation route with an atmospheric lifetime of around 1 day. At lower temperatures of interest in the ISM (11.7–177.5 K), the potential formation of NH_2_C(O)H dimers was evaluated. Thermodynamically, under equilibrium conditions, formamide would be fully converted into the dimer in that *T* range. However, the qualitative agreement of the observed increase of *k*(*T*) with computational studies on the OH + NH_2_C(O)H reaction down to 200 K let us to report, between 177.5 and 106.0 K, the following parameters commonly used in astrochemical modeling: α = (3.76 ± 0.62) × 10^–12^ cm^3^ s^–1^, β = (3.07 ± 0.11), and γ = 0. At 11.7 K, a kinetic model reproducing the experimental data indicates that formamide dimerization could be important, but the OH-reaction with the monomer would be fast, 4 × 10^–10^ cm^3^ s^–1^, and the OH-reaction with the dimer, relatively slow [(0.1–1.0) × 10^–11^ cm^3^ s^–1^]. Despite that, the impact of the gas-phase OH + NH_2_C(O)H in the relative abundances of NH_2_C(O)H in a dense molecular cloud (*T* ∼ 10 K) and after the warm-up phase in the surroundings of hot cores/corinos (here, 10–400 K) appears to be negligible.

## Introduction

The temperature conditions of molecular clouds of the interstellar medium, ISM, (∼10–100 K) and the Earth’s troposphere (∼200–298 K) are very different. However, some organic molecules are present in both environments, and their gas-phase chemistry initiated by radicals can be analogous. Formamide (NH_2_C(O)H) is one of these common organic molecules that are present in both the ISM^1^ and the Earth’s atmosphere.^2^ It has been detected in a wide variety of astronomical environments, such as high-mass star formation regions, hot corinos, and outflows associated with low-mass protostars, comets, and even in extragalactic sources.^3^ However, NH_2_C(O)H has not yet been observed in cold interstellar clouds. Its presence in clouds such as B1-b^4^ and L483^5^ is restricted to the warm gas surrounding the protostar and not to the cold extended cloud. The relevance of interstellar NH_2_C(O)H, the simplest species containing a peptide bond, is its role as a precursor of prebiotic molecules.^6−9^ Concerning the possible chemical processes producing NH_2_C(O)H, the gas-phase reaction of the amidogen radical (NH_2_) and formaldehyde (HC(O)H) was first suggested to yield NH_2_C(O)H at the very low temperatures of the ISM.^10−12^ However, this reaction was experimentally studied very recently, and it turned out to be too slow to efficiently form NH_2_C(O)H in interstellar environments.^13^ In fact, that reaction is expected to form ammonia (NH_3_) and formyl (HCO) radicals and not NH_2_C(O)H.^13^ Therefore, it is reasonable to think that the formation of NH_2_C(O)H proceeds on the surface of interstellar grains since many solid-state experiments have demonstrated the NH_2_C(O)H formation under ISM conditions.^3^ Nevertheless, the majority of these processes are also destructive, and hence, the formation mechanism of NH_2_C(O)H in the ISM is still a matter of debate.^14^ In the Earth’s atmosphere, NH_2_C(O)H has been detected in ambient air in the Kathmandu Valley (Nepal), which is prone to suffer pollution episodes,^2^ or it has been reported to be formed in situ as a product of the gas-phase photooxidation of methylamine (CH_3_NH_2_)^15^ or 2-aminoethanol (NH_2_CH_2_CH_2_OH).^16,17^

Regarding the removal routes of NH_2_C(O)H, this paper focuses on its gas-phase reaction toward hydroxyl (OH) radicals, ubiquitous in the ISM and in the Earth’s atmosphere.
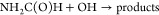
R1

To date, the gas-phase reaction R1 has only been studied experimentally around 300 K.^18,19^ Bunkan et al.^19^ computed the rate coefficient of reaction R1, *k*(*T*), in the 200–350 K range using transition state theory, observing an increase of *k*(*T*), while temperature was decreased. But there is no experimental confirmation of these results or kinetic data at temperatures below 300 K. Currently, the KIDA astrochemical database only includes the reaction of NH_2_C(O)H with C atoms, whereas the UDfA database does not even include NH_2_C(O)H reactivity. Therefore, the gas-phase kinetics of reaction R1 have to be investigated in the temperature range of interest in the dense molecular clouds and hot corinos to update astrochemical networks for use in astrochemical modeling. In addition, the extension of that kinetic study to tropospheric temperatures will allow the kinetic and photochemical data to be included in databases for use in atmospheric modeling (IUPAC, JPL-NASA) since they do not compile reaction R1 to date.

Thus, in this work, the gas-phase kinetics of reaction R1 has been studied experimentally between 11.7 and 177.5 K, covering the typical temperatures of dense molecular clouds (10–100 K) and warm-up phase in hot corinos (here, 10–400 K), and in the 263–353 K range, which overlaps the typical temperatures of the troposphere. Two experimental systems based on the Pulsed Laser Photolysis-Laser-Induced Fluorescence (PLP-LIF) technique were used to cover the entire temperature range. On one hand, the ultralow temperatures were achieved using the pulsed CRESU (French acronym for Reaction Kinetics in a Uniform Supersonic Flow) technique based on the gas expansion through a Laval nozzle. On the other hand, a slow-flow reactor (SFR) was used for the atmospheric study. The temperature in this reactor was controlled by flowing a cooled/heated liquid through its external jacket. The rate coefficients, *k*(*T*), were also determined as a function of total pressure in both experimental setups. As the extremely low pressures of the ISM cannot be achieved in the CRESU system, the pressure dependence of *k*(*T*) was studied at certain temperatures at low total pressures (*P*) between 0.11 and 1.42 mbar. In contrast, the total pressure in the SFR system was varied between 66.7 and 133.3 mbar to extend the pressure range.

## Experimental Section

In this section, the PLP-LIF experimental technique used in the kinetic studies performed in two different experimental systems over two temperature regimes (*T* = 11.7–177.5 and 263–353 K) is briefly described. The experimental systems employed to determine *k*(*T*) at the temperature conditions of the ISM (CRESU) and under conditions of the Earth’s atmosphere (SFR) have been explained elsewhere.^20−23^

The experimental conditions (*T*, *P*, flow rates, and formamide concentrations) used in both setups are different and are summarized in Tables S1 and S2 of the Supporting Information.

### Kinetic Measurements: PLP-LIF Technique

In both systems, the OH radicals were generated in the electronic ground state (X^2^Π) by PLP of an OH-precursor at 248 nm using a KrF excimer laser (Coherent, model ExciStar XS 200). The time evolution of OH (X^2^Π, *v*″ = 0), hereafter simply OH, was monitored by LIF at *ca*. 310 nm, after laser excitation at *ca*. 282 nm. The generated OH radicals were excited by the second harmonic of a tunable dye (rhodamine-6G) laser pumped by the second harmonic of a Nd/YAG laser. In the CRESU system, the excitation system consisted of a Lambda Physik dye laser (mod. Scanmate) pumped by a Continuum Nd/YAG laser (mod. Surelite III), while in the SFR system, it consisted of a LiopTech (mod. LiopStar) dye laser pumped by an InnoLas Nd/YAG laser (mod. SpitLight 1200). At the exit of the laser, the energy per pulse was ∼1 mJ pulse^–1^ at 10 Hz. At every reaction time *t*, the LIF signal, *I*_LIF_(t), was collected by a filtered photomultiplier tube (Electron Tube, model 9813B) and transferred to a gated boxcar integration unit (Standford Research System, mod. SRS250). The integrated LIF signal was recorded and processed in a computer.

In the CRESU system, the OH precursors used were H_2_O_2_ or *tert*-butyl hydroperoxide (*t*-BuOOH). In the SFR system, H_2_O_2_ and CH_3_C(O)CH_2_C(O)CH_3_ (acetylacetone, ACAC) were used at *T* > 273 K and over the *T* range studied, respectively. The gas mixture, introduced in the reactors by calibrated mass flow controllers, consisted of gaseous NH_2_C(O)H entrained by the buffer gas (*F*_buffer/NH_2_C(O)H_), the diluted OH-precursor (*F*_OH-precursor_), and an additional flow of the buffer (*F*_buffer_). The buffer gases employed in the CRESU system were He, N_2_, or Ar depending on the desired temperature as shown in Table S1, while, in the SFR experiments, helium was used as a bath gas.

Due to the very low vapor pressure of NH_2_C(O)H (0.14 mbar at 303 K^24^), it was introduced into the reactors by bubbling a flow of buffer gas through the liquid sample of NH_2_C(O)H contained in a glass bubbler. The initial NH_2_C(O)H concentration in the gas jet in the CRESU system and in the SF reactor, [NH_2_C(O)H]_0_, was varied by changing the *F*_buffer/NH_2_C(O)H_. These concentrations were measured as explained in the Methodology section. Gaseous H_2_O_2_ and *t*-BuOOH were introduced into the reactor similarly to NH_2_C(O)H by bubbling the buffer gas through a glass bubbler that contains their aqueous solution. In contrast, highly diluted mixtures for ACAC were prepared in a 10 L storage bulb (*P* = 1040 mbar, dilution factor, *f* = (1.5–2.4) × 10^–6^) to generate low initial OH concentrations since the absorption cross section of ACAC at 248 nm is extremely high (3.06 × 10^–17^ cm^2^ molecule^–1^).^25^

### Temperature Control in the Kinetic Experiments

In the pulsed CRESU, the ultracold temperatures were achieved by expanding a gas from the room-temperature reservoir, where the pressure is relatively high (*P*_res_ = 9.97–366.48 mbar) and the gas is kept at stagnation conditions, to a low-pressure chamber (*P*_ch_ = 0.12–5.20 mbar), where a very cold and supersonic gas jet is formed. The expansion is made through a Laval nozzle, which ensures a well-controlled environment of temperature and pressure. A total of six Laval nozzles were used in this work. A rotary disk with one or two holes was employed to pulse the gas at 5 Hz for the lowest temperature (*T* = 11.7 K) or at 10 Hz for the rest of temperatures (*T* ≥ 21.7 K), respectively.

In the SFR, a 200 cm^3^ jacketed Pyrex cell was used in which the temperature of the gas mixture was varied by flowing through the external jacket of the reactor cooled/heated water from a thermostatic bath (Julabo, mod. FP 50) in the 283–353 K range or a cooled ethanol/water mixture for 273 and 278 K. The gas temperature was measured using a chromel–alumel thermocouple, inserted several millimeters above the reaction zone. The total pressure in the cell was fixed to 133.32 mbar in the experiments carried out using H_2_O_2_ and 66.66 or 133.32 mbar in the experiments carried out using ACAC as the OH-precursor.

## Methodology

### Determination of the Pseudo-First-Order Rate Coefficients, *k*′

Under pseudo-first-order conditions ([NH_2_C(O)H]_0_ ≫ [OH]_0_), the temporal profile of OH in terms of the LIF intensity is described by the integrated rate eq E1.
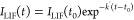
E1where *k*′ is the pseudo-first-order rate coefficient. As *I*_LIF_(*t*) is also recorded prior to the formation of OH radicals (photolysis), *t*_0_ is the reaction time from which the analysis of the decays was started. Particularly in the CRESU experiments, after the initial generation of the OH radical, a collisional rotational relaxation of OH appears, and the temporal profiles of *I*_LIF_ are given by
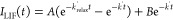
E2where *k*′_relax_ is the pseudo-first-order rate coefficient for the relaxation process by collisions with the buffer gas and/or NH_2_C(O)H, and *A* and *B* are constants related to the OH concentration rotationally excited, [OH*]_0_, and relaxed, [OH]_0_, respectively
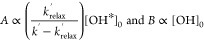
E3

**Figure 1:**
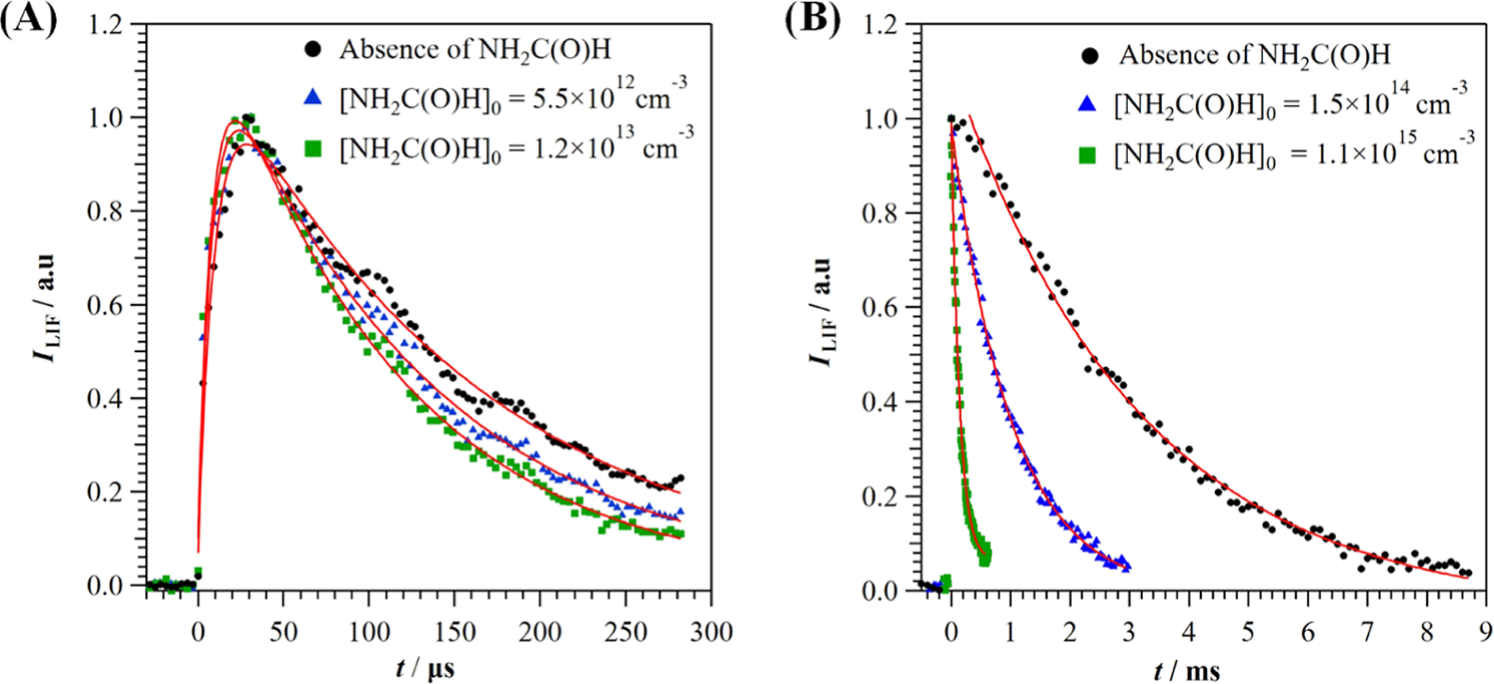
Examples of *I*_LIF_ recorded as a function of the reaction time in the presence of several initial concentrations of NH_2_C(O)H: (A) at 22.5 K for the CRESU system and (B) at 298 K for the SFR system.

**Figure 2:**
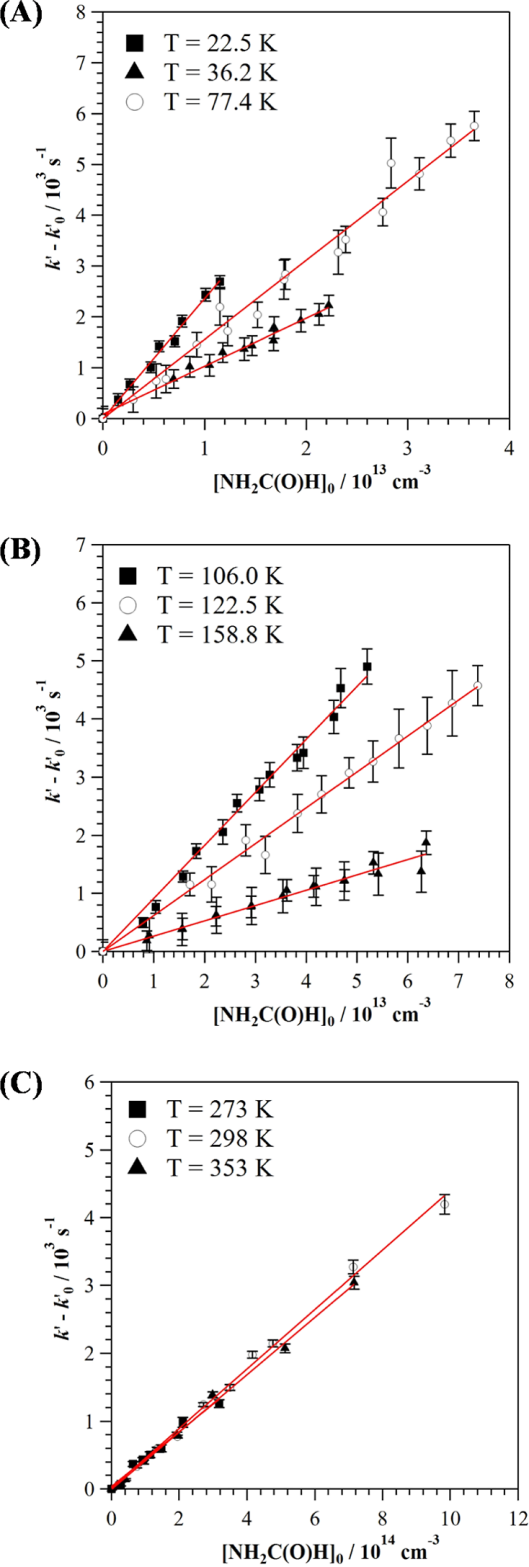
Examples of *k*′ – *k*′_0_ versus [NH_2_C(O)H]_0_ plots at (A) 22.5, 36.2, and 77.4 K; (B) 106.0, 122.5, and 158.8 K; and (C) 273, 298, and 353 K. Uncertainties in *k*′ – *k*′_0_ are the standard deviation obtained from the analysis of *I*_LIF_ decays.

In Figure 1, some examples of the *I*_LIF_ temporal profiles are shown for several initial [NH_2_C(O)H] at *T* = 22.5 K (Figure 1a) and *T* = 298 K (Figure 1b). From the analysis of the observed decays, *k*′ was obtained according to eq E1 at all temperatures. These *k*′ values are linearly related with [NH_2_C(O)H]_0_
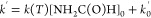
E4where *k*(*T*) is the second-order rate coefficient for reaction R1 and *k*′_0_ is the pseudo-first-order rate coefficient, which includes other loss processes of OH, such as the diffusion out of the detection zone (*T*- and *P*-dependent) or reaction with the OH-precursor. *k*(*T*) is, then, obtained from the slope of *k*′ versus [NH_2_C(O)H]_0_ plots. In Figure 2, examples of the *k*′ – *k*′_0_ vs [NH_2_C(O)H]_0_ plot are shown for several temperatures. As shown in the examples depicted in Figure S1, no influence of the OH-precursor on *k*(*T*) has been observed.

### Online Determination of [NH_2_C(O)H]_0_ Prior to the Kinetic Experiment

**Figure 3:**
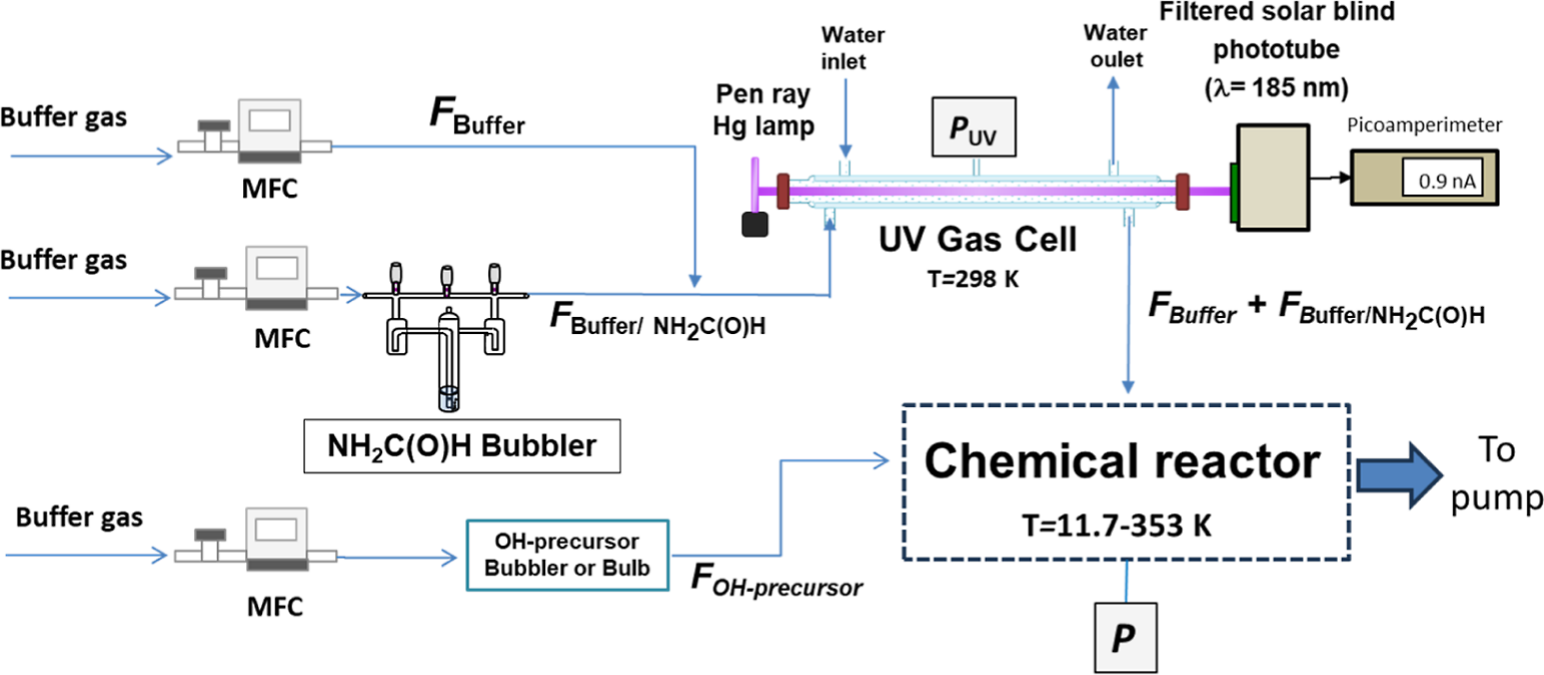
Sketch of the UV spectroscopy system to the reactor where the kinetics takes place.

The precision in the determination of *k*(*T*) depends mainly on accurate knowledge of the initial concentration of NH_2_C(O)H in the reactor, [NH_2_C(O)H]_0_ (see eq E4). For that reason, [NH_2_C(O)H]_0_ was determined during the kinetic experiments from online UV measurements at 185 nm, [NH_2_C(O)H]_UV_. These measurements were performed by flowing formamide and the buffer gas through the UV absorption cell (see Figure 3) prior to entering the reactor. *F*_OH-precursor_ was directly introduced in the reactor in both systems to avoid the interference of the absorption of H_2_O_2_ and H_2_O at 185 nm (σ_185nm_ ∼1 × 10^–18^ and ∼6.8 × 10^–20^ cm^2^ molecule^–1^, respectively).^26,27^ The 185 nm radiation was obtained from a Hg/Ar pen ray lamp (Oriel, model 6060). The gas coming from the bubbler was flown through the Pyrex UV absorption cell [path length, *l* = (93.0 ± 3.0) cm] with an outer jacket through which water from a thermostatic bath (Huber, Polystat CC1) was flown at (298 ± 2) K. The transmitted light through the absorption cell was detected by a filtered solar blind phototube (Hamamatsu Photonics, model R5764). The interference filter was centered at (185.0 ± 2.5) nm with a full-width half-maximum of 20 ± 7.5 nm (Acton Research Co., FD185-N-1D). The transmitted light intensities, both in the absence (*I*_0_) and presence of NH_2_C(O)H (*I*), were recorded by a picoamperimeter (Adler Keithley 6485) to determine the absorbance (*A*_185nm_ in base e). Finally, [NH_2_C(O)H] in the UV absorption cell, [NH_2_C(O)H]_UV_, can be determined by applying the Beer–Lambert law
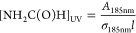
E5

The absorption cross section (in base e) of formamide at 185 nm was taken from Gingell et al.^28^ (σ_185nm_ = 7.32 × 10^–18^ cm^2^ molecule^–1^). To obtain [NH_2_C(O)H]_0_, [NH_2_C(O)H]_UV_ has to be corrected according to eq E6.
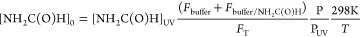
E6where *F*_T_ is the total mass flow rate through the reactor and *P*_UV_ was the pressure in the UV absorption cell (88 mbar or 166.65 mbar in the SFR system and it varies from 97 to 527 mbar in the CRESU system). The range of [NH_2_C(O)H]_0_ employed in both experimental systems for each temperature is listed in Tables S1 and S2. It is important to mention that the maximum [NH_2_C(O)H]_0_ in the CRESU system was always below 1% of the total pressure to maintain the aerodynamic characteristics of the flow, and it was not possible to increase it beyond the values appearing in Table S1 because those concentrations correspond to the situation in which *F*_buffer_ was not introduced and *F*_T_ through the reactor was only due to *F*_buffer/NH_2_C(O)H_ and *F*_OH-precursor_.

#### Chemicals

Buffer gases (He, N_2_, and Ar), all of them with 99.999% purity (Nippon Gases Europe), were used as supplied. Liquid formamide (≥99.0%, Sigma-Aldrich) was directly used without any further purification. The aqueous solution of hydrogen peroxide (50% w/w, Sharlab) was preconcentrated as previously reported.^29^*tert*-Butyl hydroperoxide (*t*-BuOOH) solution (70%, Sigma-Aldrich) was used directly without preconcentration because its vapor pressure is higher than that of H_2_O. The acetylacetone solution (≥99.0%, Sigma-Aldrich) used was diluted beforehand. When not used, both glass bubblers containing the OH-precursor were stored in a refrigerator at 4 °C to prevent thermal decomposition.

## Results and Discussion

**Figure 4:**
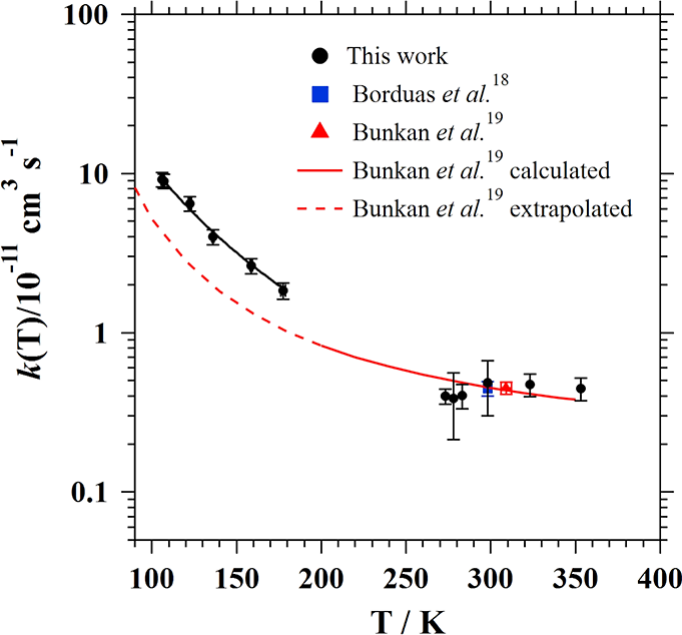
Plot of *k*(*T*) (in log form) versus *T* obtained in this work together with previously reported kinetic data between 106 and 353 K. Black solid line is the best fit of the experimental *k*(*T*) to eq E7, respectively, and solid and dashed red lines are the computed *T*-dependence of *k*(*T*) reported by Bunkan et al.^19^ and extrapolated to lower temperatures, respectively.

In kinetic studies of gas-phase reactions at ultralow temperatures, there can be a concern about the formation of reagent dimers, trimers, etc., that could affect the measurement of the rate coefficient. The binding energy of the most stable formamide dimer (*D*) has been reported to be −54 kJ/mol.^24,30^ From a thermodynamic point of view, the degree of dimerization at *T* > 273 K is negligible (see Table S3).^24^ This means that the results presented here in the 273–353 K range correspond to the OH + formamide monomer reaction. In contrast, at *T* < 177.5 K, thermodynamically, the formation of formamide dimers is expected to be favored (see Table S3). However, the amount of dimerization in the Laval nozzle expansion will be much less than the thermodynamics indicates as the system is far from equilibrium. In fact, the observed trend in the temperature dependence of *k*(*T*) between 106 and 177.5 K (Figure 4) is consistent with the computational study of Bunkan et al.^19^ that refers to the OH + formamide monomer and predicts an increase of *k*(*T*) down to 200 K. Therefore, based on the qualitative agreement between our results and those of Bunkan et al.^19^ (see below), it can be expected that little dimerization of formamide occurs in the 106–177.5 K temperature range.

Thus, the presentation and discussion of the experimental kinetic results have been divided into two temperature ranges (106–353 and 11.7–77.4 K). In addition, the kinetics of formamide dimerization at 11.7 K have been modeled and included in this section.

### Temperature Dependence of *k*(*T*) between 106 and 353 K

The pressure dependence study of *k*(*T*) for the OH-reaction with NH_2_C(O)H was carried out at 106 K (Ar or N_2_ as bath gases) between 0.73 and 2.08 mbar (see Table S1). In the 273–353 K range, the kinetic experiments were carried out at total pressures of 66.66 and 133.32 mbar, except at 273 K, when the total pressure was only 133.32 mbar. No pressure dependence of *k*(*T*) was observed at any of the investigated temperatures. Then, the rate coefficient at a single temperature is reported as the average of the individual *k*(*T*) values obtained for different OH-precursors and total pressures (see Table 1). As can be seen in Table 1, the average *k*(*T*) over the 106.0–177.5 K temperature range *k*(*T*) is on the order of 10^–10^ to 10^–11^ cm^3^ s^–1^ and increases when temperature decreases. In contrast, over the 273–353 K range, *k*(*T*) is roughly constant, within the experimental uncertainties, and on the order of 10^–12^ cm^3^ s^–1^.

**Table 1:** Summary of the Average *k*(*T*) (±2σ)^a^ as a Function of Temperature

*T*/K	*k*(*T*)/10^–11^ cm^3^ s^–1^
11.7 ± 0.7	27.7 ± 2.90^b^
22.5 ± 0.7	23.6 ± 2.51^b^
21.7 ± 1.4	21.8 ± 2.44^b^
36.2 ± 1.2	15.4 ± 1.59^b^
50.5 ± 1.6	12.5 ± 1.28^b^
52.1 ± 0.5	11.1 ± 1.20^b^
77.4 ± 1.0	9.45 ± 1.24^b^
106.0 ± 0.6	9.05 ± 0.94
107.0 ± 0.5	9.08 ± 0.92
122.5 ± 1.0	6.43 ± 0.66
136.1 ± 0.6	4.00 ± 0.44
158.8 ± 0.6	2.67 ± 0.28
177.5 ± 1.2	1.84 ± 0.22
273.15 ± 0.1	0.40 ± 0.04
278.15 ± 0.1	0.39 ± 0.17
283.15 ± 0.1	0.40 ± 0.07
298.15 ± 0.1	0.48 ± 0.18
323.15 ± 0.1	0.47 ± 0.08
353.15 ± 0.1	0.45 ± 0.07

a Statistical and 10% systematic errors.

b These values cannot be taken as the rate coefficients for the OH + formamide monomer reaction, until the contribution of dimerization is experimentally confirmed.

In Figure 4, the plot of *k*(*T*) (in log form) versus *T* is presented between 106 and 353 K together with the kinetic data previously reported in the literature.^18,19^ Clearly, two different regions are observed. In the 106.0–177.5 K range, a dramatic increase in *k*(*T*) was observed as the temperature decreases. The nature of the abrupt change in *k*(*T*) of around 5 times in 70 K can be interpreted, as for many OH-reactions, by the formation of a van der Waals prereactive complex that is sufficiently long-lived to tunnel to reaction products. Computational studies on the OH-reactivity of formamide in this temperature range are needed. The observed T-dependence of *k*(*T*) in the 106.0–177.5 K range is well described by the commonly modified Arrhenius expression [*k*(*T*) = α (*T*/300 K)^β^ exp(-γ/*T*)] used in astrochemical modeling. The best fit (black solid line in Figure 4) was obtained for γ = 0.
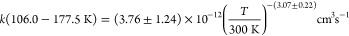
E7

Uncertainties in α and β are ±2σ statistical errors.

Between 273 and 353 K, an independent *k*(*T*), within the experimental uncertainties, was observed. We recommend the following weighted average *k*(*T*) over the investigated interval: *k* (273–353 K) = (4.11 ± 0.56) × 10^–12^ cm^3^ s^–1^, where the uncertainty is ±2σ.

### Comparison of *k*(*T*) with Previous Studies above 200 K

Table 2 lists the values of *k*(*T*) for the temperatures explored in this work and those previously reported in the literature at around room temperature for comparison purposes. Barnes et al.^31^ estimated a value of *k*(298 K) assuming it is approximately 10 times slower than the corresponding Cl reaction. Later on, Borduas et al.^18^ determined *k*(298 K) by relative kinetic measurements using H_2_O_2_ as an OH-precursor and *n*-butanol as a reference compound, while Bunkan et al.^19^ used 2-propyl nitrite as the OH-precursor and diethyl ether, toluene, and ethyl acetate as reference compounds to determine *k*(309 K). Both relative kinetic studies employed proton transfer mass spectrometry (PTR-MS) to monitor the time evolution of the formamide concentration. As Table 2 shows, the agreement, within the uncertainty limits, between our reported absolute *k*(298 K) and those from previous studies is good. Particularly, the agreement with Bunkan et al.’s^19^ results denotes that the potential acid–base reaction between H_2_O_2_ and formamide is not affecting the determination of the rate coefficient in our work. Moreover, this was confirmed by Borduas et al.^18^ who reported that the PTR-MS signal from formamide (*m*/*z* = 46) did not decay when NH_2_C(O)H was exposed to H_2_O_2_ in the dark for 15 min (see Figure 2A in Borduas et al.’s paper).

**Table 2:** Comparison of *k*(*T*) for the OH + NH_2_C(O)H Reaction with the Literature Values

*P*/mbar	*T*/K	*k*(*T*)/10^–12^ cm^3^ s^–1^	reference
67–133	273	4.00 ± 0.44^a^	this work
		5.08	Bunkan et al.^19^ (theoretical)
67–133	278	3.85 ± 1.72^a^	this work
		4.96	Bunkan et al.^19^ (theoretical)
67–133	283	4.02 ± 0.71^a^	this work
		4.85	Bunkan et al.^19^ (theoretical)
67–133	298	4.84 ± 1.78^a^	this work
1013		4.44 ± 0.46^b^	Borduas et al.^18^
		4.54	Bunkan et al.^19^ (theoretical)
1013		4	Barnes et al.^31^ (estimation)
1013	309	4.5 ± 0.4^b^	Bunkan et al.^19^ (experimental)
67–133	323	4.73 ± 0.76^a^	this work
		4.13	Bunkan et al.^19^ (theoretical)
67–133	353	4.45 ± 0.73^a^	this work
		3.75	Bunkan et al.^19^ (theoretical)

a Uncertainties in *k*(*T*) account for statistical (±2σ) and 10% systematic errors.

b Uncertainty in *k*(*T*) accounts for statistical errors (±σ).

Bunkan et al.^19^ carried out quantum chemical calculations and kinetic simulations using a master equation model to derive *k*(*T*) between 200 and 350 K. These authors reported an increase of *k*(*T*), while temperature was decreased. The computed *k*(*T*) reported by Bunkan et al.^19^ is described by the following Arrhenius expression
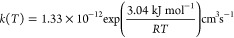
E8

We have experimentally confirmed that between 106.0 and 177.5 K, *k*(*T*) increases when decreasing temperature (Figure 4). However, although the trend is the same, the extent of this increment is not in agreement with Bunkan et al.’s^19^ extrapolated values down to 106.0 K. More computational studies are needed to confirm the observed increase in the temperature 106.0–177.5 K range. Between 298 and 273 K, *k*(*T*) from Bunkan et al.^19^ are *ca*. 20–30% higher than ours, whereas their extrapolated *k*(*T*) in the 106.0–177.5 K range are underestimated *ca*. 75–115%. Above room temperature, the computed *k*(*T*) at 323 and 353 K are *ca*. 6 and 15% lower than those determined in this work. Bunkan et al.^19^ also reported an essentially pressure-independent *k*(T) between 200 and 350 K, which is in agreement with the experimental results between 66.66 and 133.32 mbar. Considering the large experimental uncertainties in *k*(*T*) in the 273–353 K range, there is reasonable agreement.

### Kinetics of OH Radicals between 11.7 and 77.4 K

**Figure 5:**
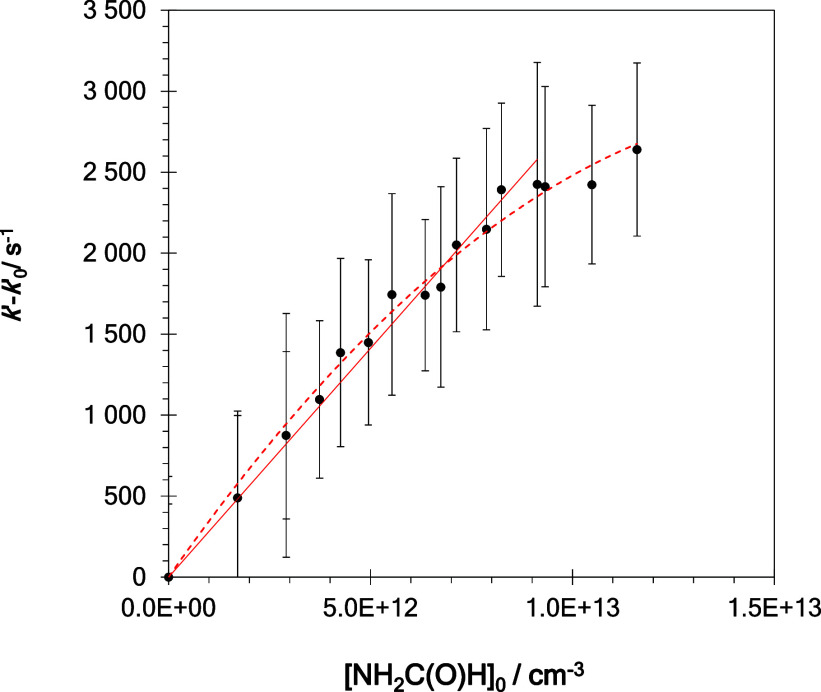
Curvature in the bimolecular plot at 11.7 K, where dimerization of formamide is more favorable. Red dashed curve is just a polynomial fit to visualize the curvature but has no physical meaning.

As shown in Table S1 of the Supporting Information, the observed loss of OH radicals at around 22 K (He as the bath gas) and 50 K (Ar or N_2_ as the bath gas) does not depend on the total pressure between 0.11 and 1.42 mbar. It is also independent of the OH-precursor used (see Figure S1). Therefore, the values reported in Table 1 at a single temperature are the average obtained for different OH-precursors and total pressures. Note that the reported values cannot be taken as the rate coefficient for the OH + formamide monomer reaction, unless the contribution of dimerization could be neglected or confirmed in experimental or theoretical kinetic studies. The only experimental evidence of the possible effect of the dimer formation (reaction R2) is a slight downward curvature in the bimolecular plot at high initial concentrations of M, [M]_0_, at 11.7 K (Figure 5).
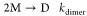
R2

The onset of dimerization was observed to be [*M*]_0_ ∼ 10^13^ cm^–3^. Below this onset, the relationship between the measured *k*′ and [*M*]_0_ is linear. In the Supporting Information, the kinetic model used to interpret the curvature in the experimental *k*′ – *k*_0_’ vs [*M*]_0_ plots at 11.7 K is presented. Four different cases were considered in which *k*_dimer_ and the rate coefficients for the OH reactions of the monomer and dimer, *k*_monomer+OH_ and *k*_dimer+OH_, were varied (Table S4). For *T* > 11.7 K, all bimolecular plots were linear, and the onset of dimerization could not be observed. For that reason, the kinetic simulation has been restricted to the lowest temperature.

As shown in the Supporting Information, case 4 is the one that better reproduces the experimental data. It is considered a very fast dimerization rate coefficient, *k*_dimer_ = 2.5 × 10^–10^ cm^3^ s^–1^, and a *k*_monomer+OH_ = 4 × 10^–10^ cm^3^ s^–1^, of the same order of magnitude than the experimental slope of the bimolecular plot (2.8 × 10^–10^ cm^3^ s^–1^). The rate coefficient for the OH-reaction with the dimer must be slower than that of the monomer to reproduce the downward curvature. *k*_dimer+OH_ was varied from 1 × 10^–12^ to 1 × 10^–11^ cm^3^ s^–1^, and no difference in the simulated bimolecular plot was observed.

For the maximum initial concentration of formamide, corresponding to the linear part of the bimolecular plot, [*M*]_0_ = 9.1 × 10^12^ cm^–3^, the kinetic model predicts a 27% of monomer conversion to dimer at the exit of the nozzle (80 μs) and 65% at the total time scale of the experiment (400 μs, including the time between the throat and the exit of the Laval nozzle). However, even though the formamide dimer could be formed at a significant rate at 11.7 K, the simulated slope in the bimolecular plot is of the same order of magnitude as the experimentally determined ones. Further studies that use detection techniques, such as rotational, IR spectroscopy, or mass spectrometry (not available in our laboratory), are needed to perform kinetic studies on the formation of formamide dimers in uniform supersonic flows and their reaction with OH radicals. This would confirm the conclusions from the present kinetic model.

### Atmospheric Implications

In the Earth’s atmosphere, the potential depletion processes of NH_2_C(O)H, apart from reaction with OH radicals, include UV photolysis in the solar actinic region (λ > 290 nm) and gas-phase reactions with other atmospheric oxidants such as NO_3_ radicals, Cl atoms, or O_3_. Tropospheric photolysis at λ > 290 nm is not likely to be an important atmospheric loss process for formamide since a negligible absorption above 225.15 nm is expected (σ_225.15nm_ = 4.12 × 10^–19^ cm^2^ molecule^–1^).^28^ Concerning the gas-phase reactivity of NH_2_C(O)H with NO_3_ and O_3_, very low upper limits for the corresponding rate coefficients have been reported at room temperature (<10^–14^ cm^3^ s^–1^ and <10^–20^ cm^3^ s^–1^, respectively).^31^ The reaction involving Cl atoms has previously been studied by Barnes et al.^31^ to be (4.5 ± 0.5) × 10^–11^ cm^3^ s^–1^ at 298 K. Considering the above-mentioned rate coefficients, the atmospheric lifetime of formamide due to Cl- and O_3_-reaction is estimated, according to eq E9, to have very low impact on the depletion of NH_2_C(O)H: *ca*. 9 months (using [Cl]_24h_ = 10^3^ cm^–3^)^32^ and 4.5 years (using [O_3_]_24h_ = 7 × 10^11^ cm^–3^),^33^ respectively.
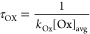
E9where *k*_Ox_ is the rate coefficient for the oxidant + NH_2_C(O)H reaction at room temperature and [Ox]_avg_ is the atmospheric average concentration for 24 or 12 h. The atmospheric lifetime of formamide due to OH-reaction ([OH]_24h_ = 10^6^ cm^–3^)^31^ derived from *k*(298 K) obtained in this work is 1.2 days, which is in agreement with that reported by Borduas et al.^18^ As the atmospheric lifetime is much lower than one year, NH_2_C(O)H is not well mixed in the troposphere. At the atmospheric boundary layer, which is about 1 km thick, the temperature would be around 288 K, assuming a surface temperature of 298 K and an adiabatic lapse rate of −9.8 °C/km. Moreover, the vertical mixing of NH_2_C(O)H in this region of the atmosphere is more important at the daytime. So, it can be transported to higher altitudes, where NH_2_C(O)H reacts with OH radicals between 298 and 273 K at a constant rate.

Regarding the nighttime depletion of formamide, it can be governed by its reaction with not only NO_3_ but also OH radicals. Even though the main atmospheric source of OH is photolytic, nighttime concentrations were measured to range between 10^4^ cm^–3^ and 10^6^ cm^–3^ in the UK, for example.^34^ Using the upper limit for the rate coefficient of the NO_3_ + NH_2_C(O)H reaction and the nighttime concentration of NO_3_ ([NO_3_]_12h_ = 5 × 10^8^ cm^–3^),^35^ the atmospheric lifetime of NH_2_C(O)H due to NO_3_ reaction is greater than 2 days, while the lifetime due to removal by OH radicals at night could range from *ca*. 8 months to 1.2 days. In any case, as the atmospheric lifetimes due to both reactive processes are larger than 12 h, the degradation of NH_2_C(O)H will be occurring both at nighttime and daytime, with the diurnal pathway being faster, as stated above.

#### Modeling the Chemistry of NH_2_C(O)H in the ISM

As stated in the Introduction, formamide is a well-known interstellar molecule detected in hot corinos and hot cores, while it has not yet been observed in cold interstellar clouds. To evaluate the impact of formamide in cold dense clouds and in hot cores/corinos, we carried out chemical modeling calculations, where it was assumed that formation of NH_2_C(O) radicals (channel R1a) is the most favorable H-abstraction channel and that the rate coefficient for reaction R1a is 4 × 10^–10^ cm^3^ s^–1^, the value obtained in the kinetic model from Supporting Information.
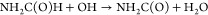
R1a

At room temperature, channel R1a is the most favorable one since the bond enthalpy in the N–H bond is higher than in the C–H bond.^18,19,31^ This was confirmed experimentally by Barnes et al.^31^ who detected at room temperature isocyanic acid (HNCO) in the presence of O_2_ as the only final product for the NH_2_C(O)H + OH reaction. Later, Nielsen et al.,^16^ Borduas et al.,^18^ and Bunkan et al.^19^ also confirmed those observations. The experimental molar yield of HNCO (17–19%)^18^ is not in agreement with the 100% yield derived from the computational studies of Borduas et al.^18^ and Bunkan et al.^19^ In any case, elementary reaction R1a was reported as the main reaction channel. HNCO was observed in interstellar space for the first time by Snyder et al.^36^ Therefore, if NH_2_C(O) radicals are the main product formed in the OH + NH_2_C(O)H reaction, one of the possible formation pathways of HNCO in the ISM could be through the radical–molecule reaction NH_2_CO + O_2_, as molecular oxygen is a very abundant molecule, with relative abundances with respect to H_2_ of about 10^–6^.^37^ However, theoretical calculations would be needed to evaluate whether this reaction is viable under the temperature conditions of the ISM.

On the other hand, the H-abstraction reaction from the amino group of formamide would yield NHC(O)H radicals (R1b), but no experimental or theoretical studies have been found to corroborate whether this channel is open at temperatures below 298 K.
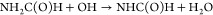
R1b

Both NH_2_C(O) and NHC(O)H radicals can contribute to the formation of more complex organic molecules by reaction with other species in the ISM. For example, the grain surface reaction CH_3_+NHC(O)H has been invoked as a source of *N*-methylformamide (CH_3_NHC(O)H), which has been tentatively detected in Sgr B2 by Belloche et al.,^38^ while the grain surface reaction between NH_2_C(O) and CH_2_OH has been also suggested to form glycolamide (NH_2_COCH_2_OH), recently detected in the molecular cloud G+0.693–0.027.^39^ The product distribution of the NH_2_C(O)H + OH reaction may vary at very low temperatures of the ISM and other reaction channels, which can be open.

### Pure Gas-Phase and Gas-Grain Astrochemical Models

**Figure 6:**
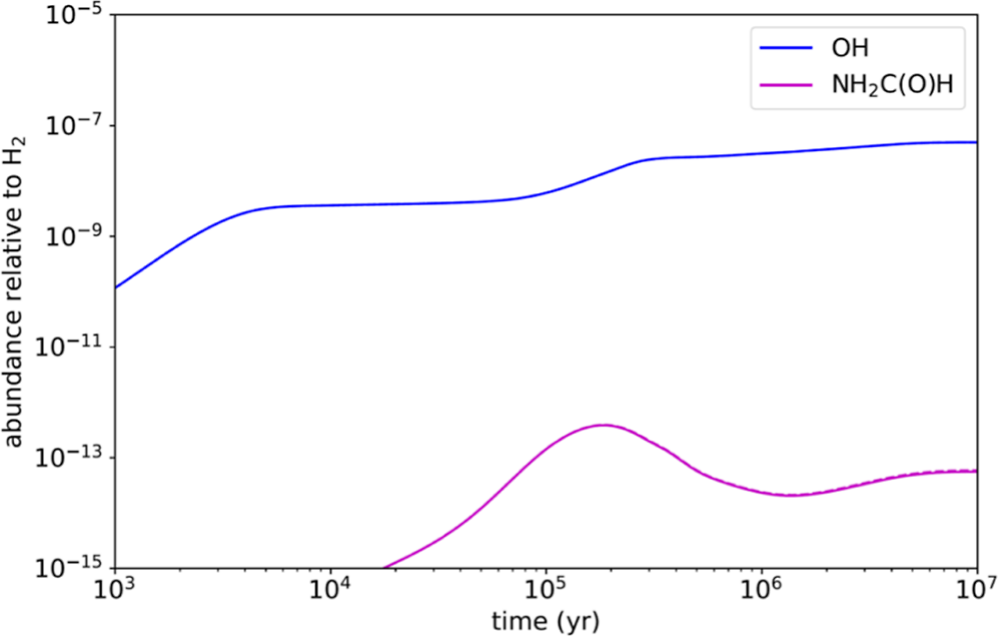
Abundances relative to H_2_ as a function of time calculated with the chemical model when including the OH + NH_2_C(O)H reaction (solid lines) with a rate coefficient of 4 × 10^–10^ cm^3^ s^–1^ and excluding it (dashed lines–not visible since it coincides with the solid lines).

In the pure gas-phase model, we adopted a gas kinetic temperature of 10 K, a volume density of H_2_ of 2 × 10^4^ cm^–3^, a visual extinction of 30 mag, a cosmic-ray ionization rate of 1.3 × 10^–17^ s^–1^, and the set of “low-metal” elemental abundances (see Agúndez and Wakelam^40^). We used the chemical network kida.uva.2014 from the KIDA database,^41^ including the reaction studied here with a rate coefficient of 4 × 10^–10^ cm^3^ s^–1^, the value obtained in the kinetic model from Supporting Information. In Figure 6, the calculated abundances of the OH radical and NH_2_C(O)H are shown as a function of time. First, it is seen that the peak abundance calculated for NH_2_C(O)H, reached at a time of 2 × 10^5^ yr, is fairly low, 3.5 × 10^–13^ relative to H_2_. This low calculated abundance is in agreement with the nondetection of formamide in cold dense clouds. Second, the plot shows modeled abundances in two cases: with the OH + NH_2_C(O)H reaction excluded (dashed lines) and with this reaction included (solid lines). It is seen that the calculated abundance of NH_2_C(O)H is essentially the same in both cases, which indicates that the reaction with OH is not a major destruction route for NH_2_C(O)H in cold IS clouds. According to the model, the most important destruction pathways of NH_2_C(O)H involve reactions with cations, such as HC(O)^+^ and H_3_O^+^.

In the gas-grain model, we use the astrochemical kinetic code MAGICKAL, which simulates the coupled gas and grain chemistry of hot cores with a three-phase treatment that considers gas-phase, grain-surface, and bulk-ice mantle chemistry. MAGICKAL uses a rate-equation based method, employing a modified-rate method when necessary.^42^ The chemical network used as the starting point is that described by Garrod and Herbst^43^ and labeled M5 by those authors. The network is extended to include reaction R1a in the gas phase using (EE7) and data from Table 1 as the rate coefficients, while the reaction was already included in the solid-phase chemistry. The grain chemistry framework includes the typical diffusive grain chemistry, as well as the nondiffusive chemistry described by Jin and Garrod^44^ and Garrod et al.,^45^ which allows for the formation of complex species on the grains, even at low temperatures. The gas-phase network includes photodissociation by external UV or the cosmic ray-induced UV field, molecular collision reactions, electronic recombination and dissociation reactions, and radiative association processes.

The physical model includes two stages: stage 1 corresponds to the cold, free-fall collapse, while stage 2 corresponds to the subsequent warming of the hot core. The free-fall collapse stage begins at a density of *n*_H_ = 3000 cm^–3^, a visual extinction of 3 mag, and a dust temperature of ∼14.7 K. The collapse continues over 10^6^ yr to a final density of 2 × 10^8^ cm^–3^, a visual extinction of 500 mag, and a dust temperature of 8 K. The gas temperature remains constant at 10 K during the collapse. In the warm-up stage, the density is held constant, while the dust and gas temperatures increase to 400 K over 2.85 × 10^5^ yr, corresponding to the “medium” warm-up time scale used in past studies. The cosmic ray ionization rate throughout both stages is held constant at 1.3 × 10^–17^ s^–1^.

**Figure 7:**
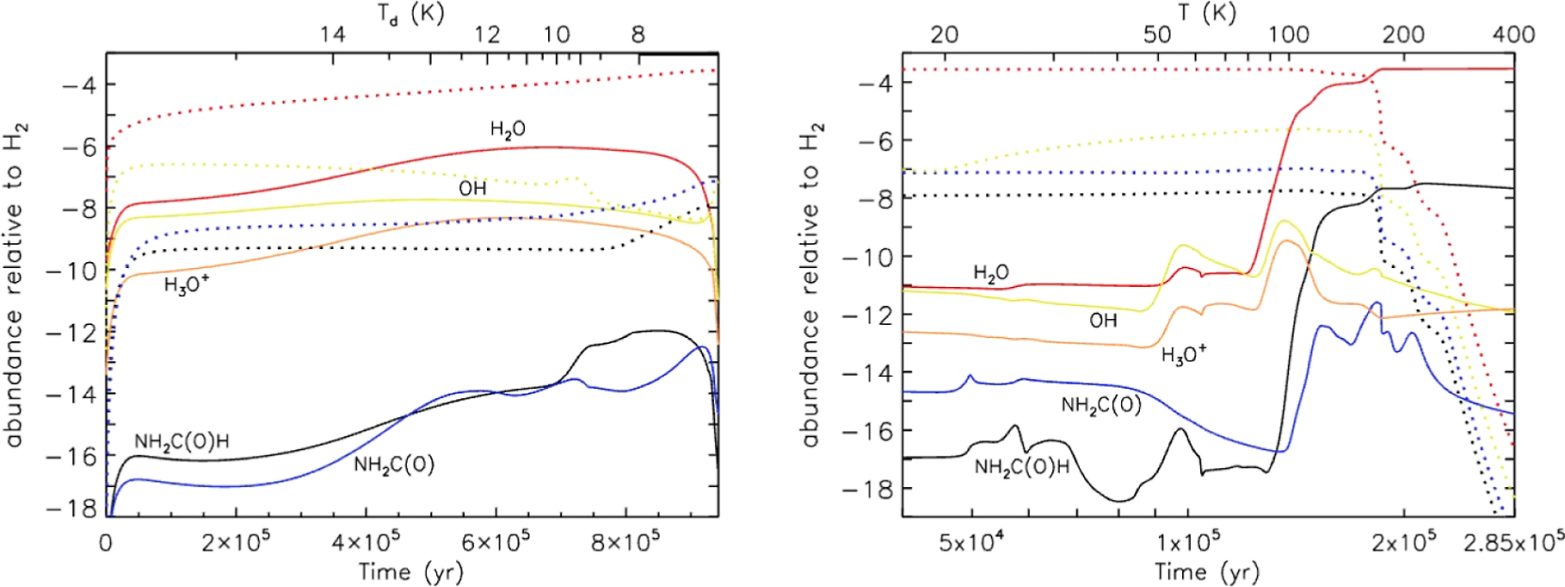
Abundances relative to H_2_ as a function of time calculated with MAGICKAL for species relevant to the OH + NH_2_C(O)H reaction. Stage 1 (collapse) is shown on the left, and stage 2 (warm-up) is shown on the right. Solid lines indicate gas-phase abundances; dotted lines of the same color indicate the species on the grain (combined surface and bulk ice).

Figure 7 shows the fractional abundances of species relevant to gas-phase destruction of NH_2_C(O)H through stages 1 and 2 with reaction R1a switched on. The peak stage 1 gas-phase abundance of NH_2_C(O)H is 1.1 × 10^–12^*n*(H_2_), which again agrees with its nondetection in cold clouds. The peak gas-phase abundance in the warm-up stage is 3.2 × 10^–8^*n*(H_2_), which agrees with formamide observational abundances in hot cores.^3^

Figure 7 in fact appears visually identical to the corresponding plot with reaction R1a switched off (not shown); the abundance of NH_2_C(O)H is (at most) only ∼3% lower with the reaction included. In stage 1, reaction R1a is not a major destruction mechanism for NH_2_C(O)H because it is more likely to react with H_3_^+^, H_3_O^+^, and HC(O)^+^, resulting in proton-transfer; even though OH has an abundance on the same order of magnitude as those ions, at 10 K the ion–neutral reactions are around an order of magnitude faster than reaction R1a. After protonation, most NH_2_C(O)H_2_^+^ recombines with an electron and is permanently destroyed. A much smaller fraction of NH_2_C(O)H_2_^+^ is deprotonated by NH_3_, returning it to NH_2_C(O)H.

In stage 2, the abundance of NH_2_C(O)H in the gas phase increases due to thermal desorption once the temperature reaches ∼100 K, and this molecule continues to be strongly thermally desorbed until ∼200 K, due to gradual release from the mixed composition of the ice mantles. Protonation and electronic dissociative recombination dominate the destruction of gas-phase NH_2_C(O)H until NH_3_ desorbs strongly from the grains at ∼120 K, allowing efficient nondestructive deprotonation. At this point, reaction R1a briefly becomes the most important destruction reaction for NH_2_C(O)H, but this destruction is dwarfed by its continuing rapid desorption from the grains. After this, cosmic ray-induced photodissociation (CRPD) tends to dominate NH_2_C(O)H destruction, while reaction R1a is the second strongest destruction mechanism of NH_2_C(O)H at temperatures below 200 K, becoming less important at higher temperatures as its rate coefficient falls.

According to the chemical models described above, the reaction between formamide and OH is not an important destruction route for NH_2_C(O)H either in cold dense clouds or in hot cores. The inclusion of reaction R1a in the MAGICKAL chemical network has only a marginal effect on the NH_2_C(O) abundance. Its gas-phase production is mostly driven by the destruction of larger species that contain the NH_2_C(O) group. In the collapse stage, the initial increase in gas-phase NH_2_C(O) production is a result of protonation of acetamide (CH_3_C(O)NH_2_) followed by the dissociative electronic recombination of the resulting protonated form, with assumed products NH_2_C(O), CH_3_, and atomic H in ∼30% of recombinations. After ∼5 × 10^5^ yr, grain temperatures decrease, and thermal desorption of CH_3_C(O)NH_2_ becomes inconsequential. At this time, the grain-surface reaction of H with HNCO, followed by chemical desorption, contributes significantly to gas-phase NH_2_C(O) abundances. In the warm-up stage of the hot-core model, gas-phase production of NH_2_C(O) is driven by the protonation and dissociative recombination of CH_2_(OH)C(O)NH_2_, as well as the CRPD of NH_2_C(O)OH; both of these species are formed on the grains prior to desorption.

## Conclusions

The present work provides the first experimental kinetic study of the loss of OH radicals in the presence of formamide below and above room temperature. Below 100 K, the potential interference of formamide dimers does not allow to presently recommend a temperature dependence expression. However, a kinetic simulation at 11.7 K provides a rate coefficient for the OH + formamide monomer of 4 × 10^–10^ cm^3^ s^–1^, very close to the experimental slope of the bimolecular plot at this temperature. Theoretical insights in the 10–100 K range are highly desirable to determine the rate coefficient for the dimerization process, on the one hand, and to calculate the rate coefficient for the OH + formamide monomer reaction, on the other hand. Above 100 K, the determination of the rate coefficient for the OH-reaction with NH_2_C(O)H as a function of temperature was carried out over two temperature regimes, *T* = 106.0–177.5 K and *T* = 273–353 K, and total pressures of 0.73–133.32 mbar. No pressure and temperature dependencies of *k*(*T*) were observed, within the experimental uncertainties in the studied range of 66.7–133.32 mbar and 273–353 K. Between 106.0 and 177.5 K, the NH_2_C(O)H + OH reaction remarkably slows down as the temperature increases according to the following modified Arrhenius expression: *k*(*T* = 106.0–177.5 K) = 3.76 × 10^–12^ cm^3^ s^–1^(*T*/300 K)^−3.07^. This equation can be used to update the astrochemical networks and to model the chemistry of formamide in the ISM by interpolating *k*(*T*). The pure gas-phase and gas-grain models assumed that the yield of NH_2_C(O) is 100% in the entire temperature range. These astrochemical models conclude that the impact of the OH + NH_2_C(O)H reaction on the observed abundances of NH_2_C(O)H is negligible. To our knowledge, NH_2_C(O) radicals have not been detected in the ISM, maybe because this formation route is not important, although its fast rate coefficient or the formation yield is lower than 100%. The detection of gas-phase reaction products at ultralow temperatures is still an experimental challenge. A reduced number of research groups is currently involved in that kind of experiments. Therefore, computational methods are excellent tools for clarifying which channel is feasible under the temperature and pressure conditions of the ISM, and further studies are needed.

Regarding the atmospheric implications, the daytime OH-reaction with NH_2_C(O)H is the main atmospheric degradation pathway of formamide, competing with its nighttime removal by NO_3_ radicals. The contributions of other atmospheric oxidants and the tropospheric photolysis are negligible. Thus, the overall atmospheric lifetime of NH_2_C(O)H due to OH-reaction is estimated to be 1.2 days, which implies that it can be degraded close to the emission source and its transport to higher altitudes.

## Supporting Information Available

Examples of pseudo-first-order rate coefficient (*k*′) vs [NH_2_C(O)H] plots at different temperatures using different OH-precursors for CRESU and SFR systems; experimental conditions (*T*, *P*, flow rates, and formamide concentrations) used in both setups; thermodynamic study of formamide dimerization; and kinetic simulation of formamide dimerization at 11.7 K (PDF)
