# Supplementary material for: Experimental Gas-Phase Removal of OH Radicals in the Presence of NH2C(O)H over the 11.7–353 K Range: Implications in the Chemistry of the Interstellar Medium and the Earth’s Atmosphere
Source: ACS Earth Space Chem. 2024 Sep 16;8(10):1970–81. doi: 10.1021/acsearthspacechem.4c00082 (PMC11492371; doi:10.1021/acsearthspacechem.4c00082)
Supplement: Supplementary file 1 — sp4c00082_si_001.pdf [file sp4c00082_si_001.pdf]

SUPPORTING INFORMATION

Experimental gas-phase removal of OH radicals in the presence of $NH_2C(O)H$ over the 11.7-353 K range: Implications in the chemistry of the interstellar medium and the Earth's atmosphere

Daniel González,[1,2] Sara Espinosa,[1,2] María Antiñolo,[1,2] Marcelino Agúndez,[3] José Cernicharo [3], Sydney Willis,[4] Robin Garrod,[4] and Elena Jiménez[1,2,*]

[1] Universidad de Castilla-La Mancha, Facultad de Ciencias y Tecnologías Químicas, Departamento de Química Física, Avda. Camilo José Cela 1B, 13071 Ciudad Real, Spain.

[2] Universidad de Castilla-La Mancha, Instituto de Investigación en Combustión y Contaminación Atmosférica, Camino de Moledores s/n, 13071 Ciudad Real, Spain.

[3] Molecular Astrophysics Group, Instituto de Física Fundamental. Consejo Superior de Investigaciones Científicas (IFF-CSIC), C/ Serrano 123, 28006, Madrid, Spain.

[4] University of Virginia, Departments of Chemistry and Astronomy, Charlottesville, Virginia 22904, USA.

*** Correspondence to:** Elena.Jimenez@uclm.es

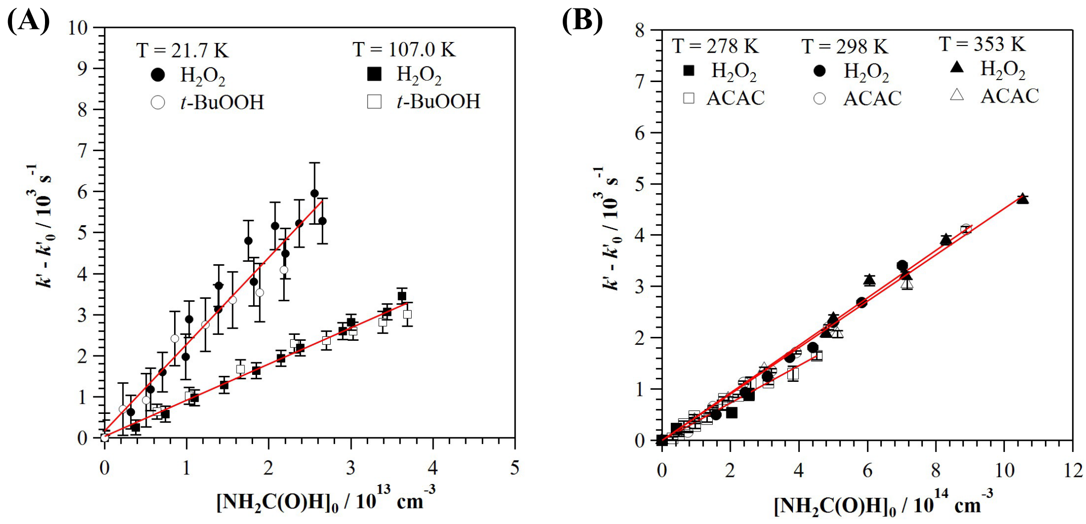

Figure S1. Examples of k'-k'_0 versus $[NH_2C(O)H]_0$ plots using different OH-precursors at: (A) 21.7 and 107.0 K (H_2O_2 or *t*-BuOOH) and (B) 278 K, 298 K, and 353 K (H_2O_2 or ACAC). Uncertainties in k'-k'_0 are the standard deviation obtained from the analysis of the I_{LIF} decays.

Table S1. Experimental conditions and individual rate coefficients (±2σ) for the OH + $NH_2C(O)H$ reaction using H_2O_2 and *t*-BuOOH as precursor at very low temperatures (11.7-177.5 K). Note that $k(T)$ at T<106.0 K cannot be taken as the rate coefficient of OH+formamide monomer until the contribution of dimerization were experimentally confirmed.

T **/ K**	***P*** **/ mbar**	***Buffer gas***	$F_{Buffer/NH_2C(O)H}$ **/ slpm** [a]	$F_{OH-precursor}$ **/ sccm** [b]	F_{buffer} **/ slpm**	**$[NH_2C(O)H]_0$ / 10^{13} cm^{-3}**	**$k'-k'_0$ / s^{-1}**	**$k(T)$ / 10^{-11} cm^3 s^{-1}**
11.7 ± 0.7	0.11	He	1.0 – 3.5	80	2.0 – 5.5	0.25 – 0.88	632 – 2340	24.4 ± 3.31 [c]
			0.9 – 5.3	200	0.0 – 5.4	0.17 – 0.91	490 - 2640	28.3 ± 2.99 [d]
22.5 ± 0.7	0.23	He	1.0 – 10.4	70	0.0 – 10.6	0.15 – 1.15	378 – 2979	23.6 ± 2.51 [d]
21.7 ± 1.4	0.51	He	0.5 – 9.9	60	0.0 – 10.2	0.23 – 2.18	692 – 4095	20.2 ± 2.78 [c]
			0.5 – 9.6	80	0.0 – 10.1	0.32 – 2.66	627 – 6359	22.6 ± 2.62 [d]
36.2 ± 1.2	0.90	He	1.5 – 9.5	20	3.8 – 13.5	0.52 – 2.84	742 – 5022	16.3 ± 2.25 [c]
			1.0 – 11.0	60	2.0 – 13.5	0.30 – 3.65	376 – 5760	15.4 ± 1.59 [d]
50.5 ± 1.6	0.11	N_2	0.3 – 1.4	5	0.0 – 1.4	0.11 – 0.82	150 – 1216	15.0 ± 1.75 [c]
			0.5 – 1.3	10	0.1 – 1.4	0.36 – 0.96	401 – 1175	12.2 ± 1.25 [d]
52.1 ± 0.5	1.42	Ar	0.1 – 1.5	10	3.5 – 5.0	0.37 – 2.28	157 – 2570	11.1 ± 1.20 [d]
77.4 ± 1.0	0.26	N_2	0.2 – 0.7	15	0.0 – 0.7	0.70 – 2.22	781 - 2223	9.45 ± 1.24 [d]
106.0 ± 0.6	2.08	Ar	0.2 – 1.4	1	0.4 – 1.9	0.93 – 4.89	938 – 3955	8.66 ± 1.09 [c]
			0.1 – 1.4	5	0.4 – 1.9	0.79 – 5.20	401 - 4899	9.12 ± 0.96 [d]
107.0 ± 0.5	0.73	N_2	0.2 – 1.1	4	0.1 – 1.2	0.64 – 3.69	637 – 3004	8.70 ± 0.99 [c]
			0.1 – 1.1	10	0.2 – 1.2	0.38 – 3.62	255 – 3451	9.14 ± 0.93 [d]
122.5 ± 1.0	1.23	Ar	0.1 – 0.7	4	0.0 – 0.8	1.62 – 7.13	1158 – 4575	6.43 ± 0.66 [d]
136.1 ± 0.8	4.74	N_2	1.0 – 5.6	20	0.0 – 5.6	1.29 – 6.04	445 – 2331	4.00 ± 0.44 [c]
158.8 ± 0.7	1.64	N_2	0.1 – 1.0	5	0.0 – 1.0	0.92 – 6.27	271 - 1373	2.47 ± 0.28 [c]
			0.1 – 1.0	5	0.0 – 1.1	0.86 – 6.36	183 – 1871	2.79 ± 0.30 [d]
177.5 ± 1.2	1.67	N_2	0.1 – 0.6	2	0.1 – 0.7	1.70 – 6.85	416 – 1037	1.81 ± 0.25 [c]
			0.1 – 0.7	8	0.0 – 0.7	1.90 – 6.60	350 – 1346	1.87 ± 0.24 [d]

[a] slpm: standard liters per minute; [b] sccm: standard cubic centimeters per minute; [c] t-BuOOH as OH-precursor; [d] H_2O_2 as OH-precursor

Table S2. Experimental conditions and individual rate coefficients (±2σ) for the OH + $NH_2C(O)H$ reaction using H_2O_2/H_2O and ACAC as precursor in the SFR system.

T / K	P / mbar	$F_{Buffer/NH_2C(O)H}$ / sccm	$F_{OH-precursor}$/ sccm	F_{He} / sccm	$[NH_2C(O)H]_0$ / 10^{14} cm^{-3}	k'-k'_0 /s^{-1}	$k(T)$ / 10^{-12} cm^3 s^{-1}
273.15 ± 0.1	133.32	30-220	2	663-900	0.57-1.90	53-700	3.93±1.34 [a]
		30-300	1	875-1200	0.27-3.22	71-1085	4.14±0.49 [a]
278.15 ± 0.1	66.66	30-150	8	238-400	0.70-3.63	346-1902	5.03±0.74 [b]
	133.32	30-300	2.5	675-1000	0.30-3.82	37-1296	3.67±0.59 [a]
			2	575-900	0.49-4.53	100-1650	3.64±0.46 [a]
		30-150	15	638-800	0.42-2.53	230-891	3.06±1.06 [b]
283.15 ± 0.1	66.66	30-150	8	138-300	2.04-8.54	595-3509	4.24±0.54 [b]
	133.32	30-300	2	576-900	0.26-5.94	62-2244	3.67±0.27 [a]
		30-220	2	762-1000	0.28-4.12	110-1618	3.89±0.42 [a]
		30-150	10	238-400	1.53-7.87	430-3165	4.29±0.46 [b]
298.15 ± 0.1	66.66	10-100	15	400-500	1.09-5.94	256-2623	4.66±0.52 [b]
		70-200	8	100-300	3.66-10.29	2171-6085	5.54±0.74 [b]
	133.32	30-300	2.5	330-650	0.75-9.83	330-4195	4.39±0.20 [a]
			2	226-550	0.75-8.88	164-4111	4.69±0.20 [a]
		10-100	15	400-500	0.13-3.14	77-1859	5.80±0.33 [b]
		50-280	18	100-400	1.84-12.69	905-6830	5.47±0.32 [b]
		50-300	10	367-690	1.58-9.07	496-4531	5.13±0.33 [b]
323.15 ± 0.1	66.66	80-280	20	410-710	0.74-8.26	882-4574	4.89±0.49 [b]
		10-250	10	130-400	0.86-8.75	540-4509	5.14±0.28 [b]
	133.32	30-300	1	875-1200	0.13-2.65	163-1222	4.59±0.97 [a]
			2	677-1000	0.30-2.38	124-992	4.32±0.60 [a]
		30-220	5	363-600	0.41-4.06	220-1799	4.55±0.64 [a]
			3	387-710	0.42-6.88	242-3426	4.90±0.27 [b]
353.15 ± 0.1	66.66	65-155	9	45-200	5.00-8.32	2295-3916	4.76±0.48 [b]
		10-250	8	130-400	0.18-6.70	175-3798	5.66±0.15 [b]
	133.32	30-300	2	327-650	0.42-7.16	139-3042	4.23±0.20 [a]
			3	277-600	0.55-5.72	165-2333	4.12±0.10 [a]
		80-200	15	100-220	4.80±10.54	2081-4692	4.41±0.44 [b]
		30-300	11	277-600	0.43-7.22	160-3499	4.72±0.29 [b]
			3	367-600	0.63-4.17	163-2304	5.67±0.60 [b]

[a] ACAC as OH precursor; [b] H_2O_2/H_2O as OH-precursor.

Thermodynamic aspects of formamide dimerization

Using the standard enthalpy (ΔH^0) and the standard entropy (ΔS^0) of the dimerization equilibrium, -53.05 kJ mol^{-1} and -156.6 J mol^{-1} K^{-1} (Emel'yanenko *et al.,* 2011), respectively, the equilibrium constant (K_p) for dimerization can be calculated as a function of temperature.

$$2\,M \rightleftarrows D \qquad\qquad K_p$$

The degree of dimerization α, defined as the fraction of molecules that are H-bonded can be calculated from K_p listed in Table S3, defined as (Prausnitz *et al.*, 1998):

$$K_p = \frac{\alpha(1 - {}^{\alpha}/_{2})P^0}{2(1 - \alpha)^2 P}$$

where P^0 = 101.325 kPa and P is taken as the partial pressure of formamide in the jet in kPa calculated from $[M]_0$. The equilibrium concentrations of the formamide monomer, $[M]_e$, and dimer, $[D]_e$, are calculated as:

$$[M]_e = [M]_0\,(1-\alpha)$$

$$[D]_e = \alpha/2\,[M]_0$$

As shown in Table S2, under our experimental conditions, the conversion of monomer into dimer in the equilibrium is almost complete between 106 and 177.5 K, but lower than 1% at temperatures above 273 K. So, from the thermodynamic point of view the formation of formamide dimers is expected to be negligible at T>273 K. Below that temperature, K_p is much higher, being the equilibrium totally shifted to the formation of the dimer.

Table S3. Equilibrium dimerization constant, dimerization degree, equilibrium concentrations of formamide monomer (M) and formamide dimer (D) and % of conversion monomer-dimer for the experimental conditions of this work.

T/ K	K_p	n/ 10^{17} cm^{-3}	$[M]_0$/ 10^{13} cm^{-3}	P / kPa	α(T,P)	$[M]_e$/ cm^{-3}	$[D]_e$/ 10^{13} cm^{-3}	% Conversion 2 M $\rightarrow$ D
106.0	9.67×10^{17}	1.40	5.20	7.61×10^{-5}	1.00	3.05×10^{7}	2.60	100
107.0	5.51×10^{17}	0.49	3.70	5.46×10^{-5}	1.00	3.39×10^{7}	1.85	100
122.5	2.91×10^{14}	0.72	7.10	1.20×10^{-4}	1.00	1.91×10^{9}	3.55	100
136.1	1.60×10^{12}	2.50	6.00	1.13×10^{-4}	1.00	2.25×10^{10}	3.00	100
158.8	1.96×10^{9}	0.74	6.40	1.40×10^{-4}	0.99	6.14×10^{11}	3.17	99
177.5	2.85×10^{7}	0.67	6.90	1.69×10^{-4}	0.93	4.99×10^{12}	3.20	93
273.0	98.51	35.4	32.0	1.21×10^{-3}	0.002	3.19×10^{14}	0.044	0.2
353.0	90.45	27.3	105	5.14×10^{-3}	0.009	1.04×10^{15}	0.477	0.9

Evaluation of the formation of formamide dimer at 11.7 K by kinetic simulations

The concentrations of M and D in the jet obviously depend on the rate coefficient of dimerization and the rate coefficients of the OH-reactions. To check if the dimerization kinetics could affect the monomer concentration, essential parameter to accurately determine the bimolecular rate coefficient, the OH temporal profiles were simulated using FACSIMILE program in the worst case (11.7 K, where the timescale of the experiment is ca. 300 μs), where the formamide dimerization process would be more favorable. Note that, at higher temperatures, the following reasoning becomes even more in favor of the presence of formamide as a monomer, since the rate coefficients used in the simulation are expected to be lower.

The OH temporal profiles were simulated assuming the following simple reaction scheme:

Formamide dimerization:	$2\,M \rightarrow D$	k_{dimer}
OH-reaction of the monomer:	$M + OH \rightarrow P1$	$k_{monomer+OH}$
OH-reaction of the dimer:	$D + OH \rightarrow P2$	$k_{dimer+OH}$

The reaction of OH with the OH-precursor and diffusion are not included to directly derive k'-k_0' at each $[M]_0$ from the analysis of the OH profiles, as done in the analysis of the experimental data. The simulated k'-k_0' were plotted against $[M]_0$ to derive the "apparent" simulated rate coefficient, k_{sim}:

$$(k'\text{-}k_0')_{simulated}=k_{sim}[M]_0$$

In all simulations, the initial concentration ranges were:

$$[M]_0 = (1.7\text{-}15)\times10^{12}\ cm^{-3}$$

$$[D]_0 = (0.34\text{-}3.0)\times10^{10}\ cm^{-3}$$

The rate coefficients assumed in the simulations are summarized in **Table S4.** We have assumed a dimerization rate coefficient similar to that for benzene at 14.9 K (Hamon *et al.*, 2000). For our gas density at 11.7 K (6.88×10^{16} cm^{-3}), k_{dimer} is taken as 2.5×10^{-10} cm^3 s^{-1} and the initial concentration of OH radicals $[OH]_0$ was estimated to be lower than 10^{10} cm^{-3}. So, we consider that value as an upper limit.

The following cases were considered in the simulation:

- CASE 1: The dimerization process is fast and the monomer is not reacting with OH.
- CASE 2: The dimerization process is fast and D is not reacting with OH.
- CASE 3: The dimerization process is slower and M reacts with OH much faster than D.
- CASE 4: The dimerization process is fast, M reacts with OH much faster than D and $k_{OH+monomer}$ is adjusted to match the experimental data.

Table S4. Summary of the rate coefficients used in the simulation and the obtained simulated rate coefficient, k_{sim}*.

	k_{dimer}/ $cm^3\ s^{-1}$	$k_{monomer+OH}$/ $10^{-10}\ cm^3\ s^{-1}$	$k_{dimer+OH}$/ $10^{-10}\ cm^3\ s^{-1}$	k_{sim}/ $10^{-10}\ cm^3\ s^{-1}$
**Case 1**	2.5×10^{-10}	-	2.8	0.56
**Case 2**	2.5×10^{-10}	2.8	-	1.7
**Case 3**	5.0×10^{-11}	2.8	0.01-0.1	2.5
**Case 4**	2.5×10^{-10}	4.0	0.01-0.1	2.4
**Case 4** Upper limit	2.5×10^{-10}	5.0	0.01-0.1	3.1
**Case 4** Lower limit	2.5×10^{-10}	3.0	0.01-0.1	1.8

* Result of the fit, neglecting the curvature and considering only the linear part of the simulated bimolecular plots.

CASE 1: The dimerization process is fast and the monomer is not reacting with OH.

If we consider that the observed OH reactivity only comes from the formamide dimer, the results of the simulation are:

- The OH profiles (in *ln* form) are not linear, which is not consistent with the experimental observations.
- The simulated bimolecular plot (**Figure S2,** blue squares) is slightly curved upwards, which is not consistent with the experimental plots (**Figure S2,** black circles). Dashed curves in the figure are just a mathematical polynomial fit just to visualize the curvature but have no physical meaning.
- The pseudo-first order rate coefficients are largely underestimated (**Fig. S2**).

Therefore, under the simulated conditions of CASE 1, formamide dimers are not likely to be responsible of the observed OH-decays. Moreover, the OH temporal profiles are not exponential, even at low monomer concentrations.

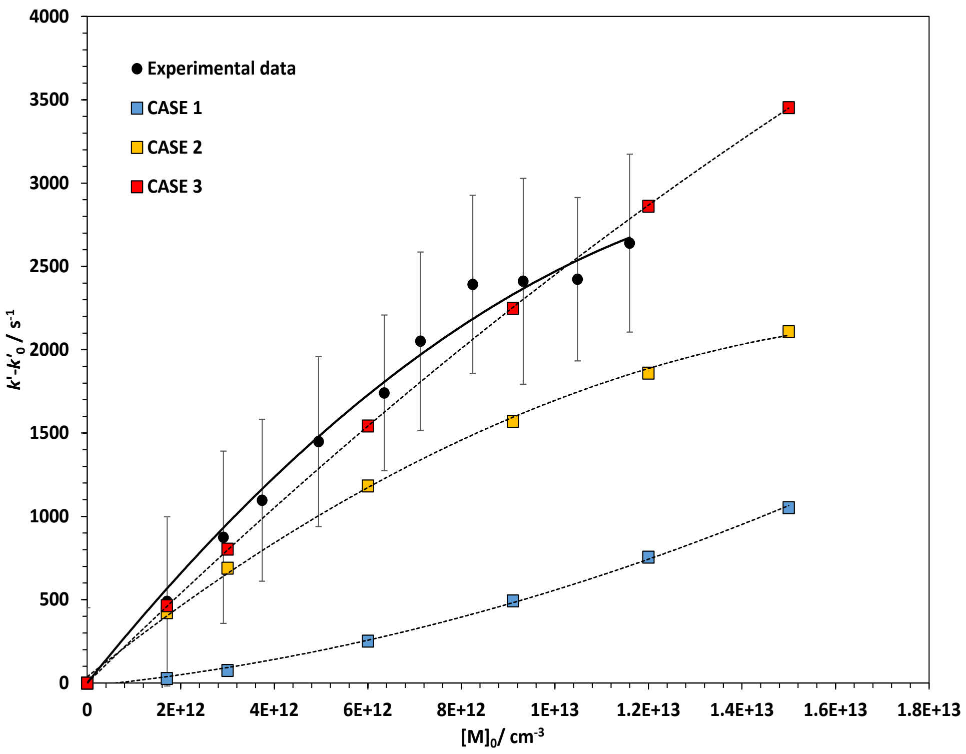

Figure S2. Comparison between experimental and simulated bimolecular plots for Cases 1, 2 and 3.

CASE 2: The dimerization process is fast and D is not reacting with OH.

In this case, we consider that the dimerization process continues at low temperatures and that the OH reactivity only comes from the monomer with the experimental rate coefficient ($k_{OH+monomer}$=2.8×10^{-10} cm^3 s^{-1}). The results of the simulation are:

- The OH profiles (in *ln* form) are linear at low monomer initial concentrations, but they are slightly curved at high monomer concentrations.
- The bimolecular plot is slightly curved downwards at high monomer initial concentrations, as observed experimentally (**Figure S2**). However, the pseudo-first order rate coefficients are somehow underestimated, probably due to all the assumptions and unknowns.

Therefore, under the simulated conditions of CASE 2, when the monomer is the sole responsible species for the OH-decays, the downward curvature in the bimolecular plots is well reproduced. However, the simulated pseudo-first order coefficients are underestimated and the onset for dimerization is lower than experimentally observed.

CASE 3: The dimerization process is slower and M reacts with OH much faster than D.

This case assumes a slower rate coefficient for the dimerization and that the monomer reacts at the experimental rate coefficient ($k_{OH+monomer}$=2.8×10^{-10} cm^3 s^{-1}) and the dimer reacts much slower ($k_{OH+dimer}$=(0.1-1.0)×10^{-11} cm^3 s^{-1}). For this range of reactivity, the effect of $k_{OH+dimer}$ in the bimolecular plot in that range is negligible. The results of the simulation are:

- The OH profiles are linear over the entire timescale of the experiment.
- The bimolecular plot is linear over the entire experimental monomer concentration range and beyond, which is not experimentally observed (**Figure S2**).
- The downward curvature in the bimolecular plot is not reproduced.

Therefore, under the simulated conditions of CASE 3, the downward curvature in the bimolecular plots is not reproduced, indicating that the dimerization rate coefficient is not on the order of 10^{-11} cm^3 s^{-1}.

CASE 4: The dimerization process is fast, M reacts with OH much faster than D and $k_{OH+monomer}$ is adjusted to generate the best match to the experimental data.

This case assumes $k_{OH+dimer}$=(0.1-1.0)×10^{-11} cm^3 s^{-1} and $k_{OH+monomer}$ has been changed between 3.0×10^{-10} cm^3 s^{-1} (lower limit in **Figure S3**) and 5.0×10^{-10} cm^3 s^{-1} (upper limit in **Figure S3**) to reproduce the experimental data. The results of the simulation are:

- The OH profiles are linear over the entire timescale of the experiment.
- The downward curvature in the bimolecular plot and the onset of dimerization is well reproduced for $k_{OH+monomer}$=4.0×10^{-10} cm^3 s^{-1} (**Fig. S3**).
- The value of $k_{OH+monomer}$=4.0×10^{-10} cm^3 s^{-1} is on the same order of magnitude that the experimentally measured (2.8×10^{-10} cm^{3}s^{-1}). Although the difference in $k_{OH+monomer}$ is about 40%, the uncertainties in the model can be the source of that difference.

Therefore, under the simulated conditions of CASE 4, the downward curvature in the bimolecular plots and $k_{OH+monomer}$ are satisfactorily reproduced, indicating that the dimerization rate coefficient is on the order of 10^{-10} cm^3 s^{-1} and that the formamide dimer is reacting much slower than the monomer.

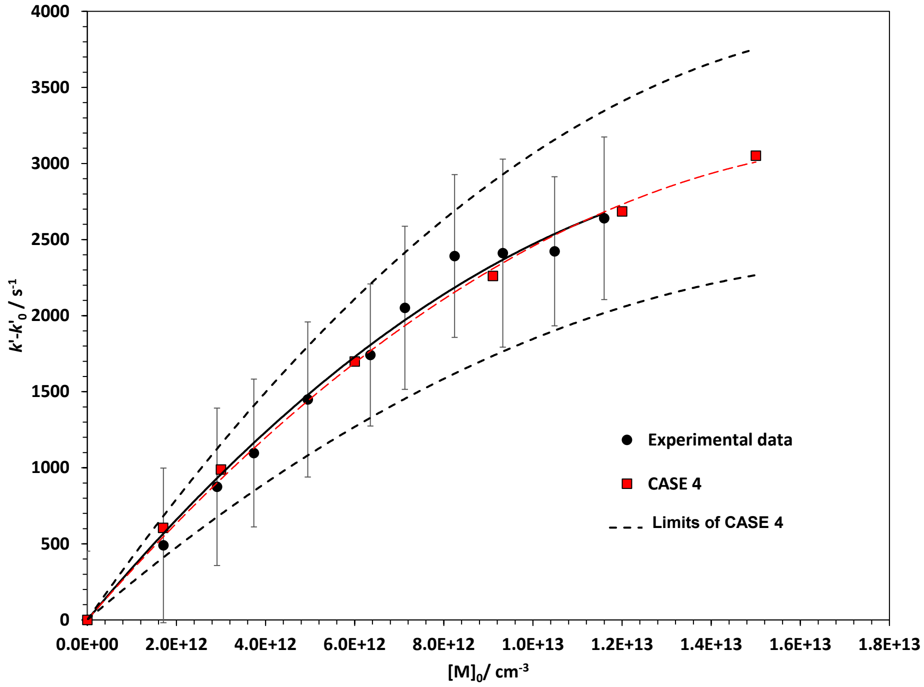

Figure S3. Comparison between experimental and simulated bimolecular plots for Case 4.

In conclusion, even though we cannot ensure without any doubt that formamide dimers are not formed at low temperatures under our experimental conditions, based on the simulations carried out we can conclude that, if formamide dimers are formed in the jet, the determined rate coefficients in this work do not correspond to the OH-reaction with the dimer.

References

Emel'yanenko, V. N.; Verevkin, S. P.; Varfolomeev, M. A.; Turovtsev, V. V.; Orlov, Y. D. Thermochemical Properties of Formamide Revisited: New Experiment and Quantum Mechanical Calculations. *J. Chem. Eng. Data* **2011**, *56* (11), 4183–4187.

Hamon, S.; Le Picard, S. D.; Canosa, A.; Rowe, B. R.; Smith, I. W. Low temperature measurements of the rate of association to benzene dimers in helium. *J. Chem. Phys.* **2000**, 112(10), 4506-4516.

Lucas, B.; Grégoire, G.; Lecomte, F.; Reimann, B.; Schermann, J. P.; Desfrançois, C. Infrared spectroscopy of mass-selected neutral molecular systems without chromophore: the formamide monomer and dimer. *Mol. Phys.* **2005**, *103*, 1497-1503.

Prausnitz, J. M.; Lichtenthaler, R. N.; De Azevedo, E. G. Molecular Thermodynamics of Fluid-Phase Equilibria; *Pearson Prentice Hall*, 1998.
